# Genotype Shift of Malaysian Porcine Circovirus 2 (PCV2) from PCV2b to PCV2d within a Decade

**DOI:** 10.3390/ani12141849

**Published:** 2022-07-21

**Authors:** Chew Yee Tan, Roongroje Thanawongnuwech, Siti Suri Arshad, Latiffah Hassan, Michelle Wai Cheng Fong, Peck Toung Ooi

**Affiliations:** 1 Faculty of Veterinary Medicine, Universiti Putra Malaysia, UPM Serdang, Selangor 43400, Malaysia; tanchewyee.vivian@gmail.com (C.Y.T.); suri@upm.edu.my (S.S.A.); latiffah@upm.edu.my (L.H.); michelle@upm.edu.my (M.W.C.F.); 2 Department of Veterinary Pathology, Faculty of Veterinary Science, Chulalongkorn University, 39 Henri Dunant Road, Pathumwan, Bangkok 10330, Thailand; roongroje.t@chula.ac.th

**Keywords:** porcine circovirus type 2 (PCV2), porcine circovirus type 2a (PCV2a), porcine circovirus type 2b (PCV2b), porcine circovirus type 2d (PCV2d), genotype shift, pigs, Malaysia

## Abstract

**Simple Summary:**

This study aims to provide an updated Malaysian porcine circovirus 2 (PCV2) situation after a knowledge gap of one decade. Molecular detection rates of 83.78% and 83.54% at farm and sample population level were reported, close to previous publication. However, an obvious genotype shift from genotype PCV2b to PCV2d was revealed. Substitution rate for PCV2 *cap* gene sequences in this study was estimated at 1.102 × 10^−3^ substitutions/site/year, in agreement with the high substitution rate expected from PCV2 strains. Phylogenetic clustering pattern according to the year of sample origin was observed, suggesting possible nucleotide mutation occurring over time. Concurrent circulation of different PCV2 strains within one farm and within a single individual were also observed. This study also reports detection of PCV2 antigen across all production age groups from fetuses to sows; in abattoir lung samples from clinically healthy finishers; and in the wild boar population roaming Peninsular Malaysia. These observations of high molecular detection rates in farms, clinically healthy abattoir samples and in the wild boar population; and most importantly, a new wave of genotype shift from PCV2b to PCV2d—warrant further attention on the Malaysian PCV2 situation pertinent to the control and management strategy applicable to local swine farming.

**Abstract:**

This paper aims to update the molecular status of porcine circovirus 2 (PCV2) in Malaysia. Firstly, the molecular detection rate of PCV2 in farm and sampled pig population were reported to be 83.78% (31/37 farms) and 83.54% (66/79 pigs) positive for PCV2, respectively. PCV2 was detected across all age groups, from fetuses, porkers to sows. Co-detection of PCV2 and PCV3 antigens was also reported at a rate of 28.77% (21/73). Secondly, PCV2 antigen was also detected in Malaysian abattoir lung samples: 18 out of 19 (94.74%) samples originating from clinically healthy finishers were tested positive. Further, this is the first study to confirm the circulation of PCV2 in the wild boar population roaming Peninsular Malaysia, where 28 out of 28 (100%) wild boar lung samples were found positive. One decade earlier, only genotype PCV2b was reported in Malaysia. This most recent update revealed that genotypes PCV2a, PCV2b and PCV2d were present, with PCV2d being the predominant circulating genotype. PCV2 *cap* gene nucleotide sequences in this study were found to be under negative selection pressure, with an estimated substitution rate of 1.102 × 10^−3^ substitutions/site/year (ssy).

## 1. Introduction

Porcine circovirus 2 (PCV2) is considered ubiquitous in the swine population. In the history of circoviruses of swine, porcine circovirus type 1 (PCV1) was first discovered in 1974 as an apathogenic cell culture contaminate [1]. Two decades later, the first clinical outbreak of postweaning multisystemic wasting syndrome (PMWS) was reported from December 1994 to March 1996, with a characteristic presentation of unthriftiness, jaundice, respiratory distress, diarrhea and increased postweaning mortality rate [2,3]. PCV2 antigen was detected in multiple organs from postweaning pigs with PMWS-associated clinical signs and pathological changes [4]. The term porcine circovirus-associated diseases (PCVAD) was coined by the American Association of Swine Veterinarians (AASV) in March 2006 to classify the complex PCV2-related multi-factorial diseases under one umbrella term [5]. Another two decades from the report of PCV2, a genetically divergent circovirus, the designated porcine circovirus type 3 (PCV3), was uncovered through metagenomic sequencing when investigating a case of increased sow mortality rate and decreased conception rates with presenting dermal and renal lesions, suggestive of porcine dermatitis and nephropathy syndrome (PDNS) [6]. Most recently in 2019, porcine circovirus type 4 (PCV4) was reported to be the newest PCV member. The index herd was reported to show severe clinical signs of respiratory disease, enteritis and PDNS [7]. As with other members of the family Circoviridae and genus Circovirus, PCV2 is an icosahedral, non-enveloped virus that assembles into a single-stranded ambisense circular genome [8]. With a genome length ranging from 1766 bp to 1769 bp, PCV2 is larger than PCV1 (1758–1760 bp); smaller than PCV3 (1999–2001 bp) and PCV4 (1770 bp) [6,7,9,10]. Despite their different lengths, three common major open reading frames (ORFs): ORF1, ORF2 and ORF3, have been recognized in the genomes of all porcine circoviruses. Respectively, ORF1, ORF2 and ORF3 are responsible for encoding proteins involved in viral genome replication (Rep and Rep′), the capsid protein (Cap), and possibly in pathogenesis [11,12,13,14]. Based on a more recent genome-wide, pairwise identity-based genome identity calculation method, the pairwise identity cut-off for circovirus species fell between 78% to 80%; with PCV1 and PCV2 sharing 79% of a genome-wide pairwise identity. PCV1 and PCV2 shares 86% and 65% similarity of amino acid (aa) identity in their ORF1 and ORF2, respectively [15,16]. Considering the fact that PCV1 and PCV2 have always been considered as two separate species as supported by their distinct biological differences in terms of pathogenicity, the species demarcation for circoviruses is delineated at 80% [17]. PCV3 and PCV4 show lower identity similarity. Compared to PCV2, PCV3 shows only <50% of overall nucleotide (nt) identity; 48% and 26–36% aa identity of ORF1 and ORF2, respectively [6,18]. Respective to the genomes of PCV1, PCV2 and PCV3, PCV4 showed 50.3%, 51.5% and 43.2% nt similarities [7]. The *Cap* gene was focused in this study, given that it is known to demonstrate the highest genetic variation when compared to other regions of the entire PCV2 genome sequence [11,19]. By observing the differences over nucleotide sequences of *cap* genes, the population diversity and genetic variation of PCV2 strains could be assessed [20].

After 16 years of vaccination effort against PCV2, although the virus is still circulating in production systems, PCV2 positive PCVAD cases are reported at a declining rate [21]. Being a single-stranded DNA virus with high nucleotide substitution rates comparable to RNA viruses, genome mutation of PCV2 is expected to be high [22]. Indeed, nucleotide polymorphism of PCV2 has been characterized in one decade post its first discovery [23]. Since then, more novel genotypes and phylogenetic clusters of PCV2 continued to be discovered. To date, eight PCV2 genotypes, PCV2a through PCV2h, have been described [24]. In Malaysia, the presence of PCV2 and PCV3 in commercial swine herds has been confirmed [25,26]. The circulating PCV2 strains were determined to 1767 bp in length and clustered as a PCV2b genotype. This paper aims to update the molecular characterization of PCV2 strains circulating in both Malaysia commercial swine herds and in wild boar population. Considering the continuing economic impact of PCV2 on the swine farming industry worldwide, there is a need to update the genetic characteristics of current Malaysian PCV2 strains as part of the collective effort in optimizing PCV2 disease management and control.

## 2. Materials and Methods

### 2.1. Animals

A total of 111 lung and inguinal lymph nodes samples from the authors’ previous PCV3 sample collection were included in this study. Samples’ origin and collection method were as previously described [26]. Briefly, 37 commercial intensive farms in Penang, Perak; Selangor; Melaka and Johor states representing northern; central and southern regions of Peninsular Malaysia, respectively, were involved. The farms were run on a semi-closed house, farrow-to-finish system, with a farm size ranging from 200 to 1500 sows. Samples comprised of archived lung samples from year 2016 to year 2017, farm clinical samples, and abattoir lung samples collected from year 2018 to year 2021. Apart from that, 28 lung samples from the wild boar population in Peninsular Malaysia collected in year 2019 were also included in this study.

### 2.2. Molecular Detection of PCV2

DNA extraction was performed using the DNeasy Blood & Tissue Kit extraction kit (Qiagen, Hilden, Germany) in accordance to the manufacturer’s instructions. Conventional PCR was performed using MyTaq™ Red Mix 2X (Bioline, London, UK). Published primers, FortF–5′–CTTTTTTATCACTTCGTAATG–3′ and FortR–5′–CGCACTTCTTTCGTTTTC–3′, were used to amplify the capsid (*cap*) region of PCV2 [27]. Briefly, 12.5 μL of the Taq DNA polymerase master mix and 1.0 μM each from the primer pair were used in a 25 μL total PCR reaction volume. Cycling conditions of the conventional PCR were as described by Fort et al. (2007) [27]. PCR products were stained using RedSafe™ nucleic acid staining solution (iNtRON Biotechnology, Seongnam, Korea) and analyzed by agarose electrophoresis. Expected product length was a 760 bp representing the PCV2 *cap* region spanning from position nt 998–1757. Samples that showed a positive band at the between 700–800 bp region as marked by GelPilot 100 bp Plus Ladder (Qiagen, Germany) were further sequenced (Macrogen, Seoul, Korea). Since the samples were obtained from a PCV3 study, the relative risk (RR) and Odds ratio (OR) of co-infection with PCV3 were computed as well, using IBM SPSS Statistics for Windows v23 software [28].

### 2.3. Pairwise Sequence Comparison (PASC) Analysis

Seventy-nine samples positive for PCV2 *cap* gene by PCR detection were sequenced to confirm the identity of the PCV2 *cap* gene nucleotide sequences: 51 sequences from domestic pigs and 28 sequences from wild boars. Sequence assembly and multiple sequence alignment were generated with the MUSCLE algorithm using MEGA v7.0.26 software [29]. The resulting sequences were analyzed using NCBI Nucleotide-BLAST^®^ for a final identity confirmation as PCV2 by comparing the nucleotide similarity with reference to PCV2 sequences deposited in the GenBank [30]. Other 162 PCV2 *cap* gene nucleotide sequences were downloaded from GenBank to be used as reference sequences in the subsequent phylogenetic analysis. The sequences were ensured to be well-aligned, in correct reading frame without degenerate nucleotides or indels, of different country origin and representative of clusters PCV2a through PCV2h. Pairwise distance estimation based on p-distance nucleotide substitution model with 1000 bootstrap replicates was calculated across the entire set of 241 PCV2 *cap* gene nucleotide sequences, using the same MEGA v7.0.26 software. Based on the pairwise distance estimation data matrix, pairwise sequence comparison (PASC) analysis was performed by constructing a p-distance/frequency histogram using Microsoft Excel 2019 to determine possible cut-off values for distinguishing different PCV2 genotypes.

### 2.4. Phylogenetic Analysis and Genotyping

A neighbor-joining (NJ) phylogenetic tree based on a p-distance nucleotide substitution model with 1000 bootstrap replicates was constructed to analyze the 241 PCV2 *cap* gene nucleotide sequences dataset, similarly with MEGA v7.0.26 software, as described previously [29]. Resulting phylogenetic clustering was used to classify the PCV2 sequences into genotypes PCV2a to PCV2h. The phylogenetic-based genotype classification was tallied with genotype classification systems proposed by Franzo et al. (2015) and Franzo et al. (2018) [24,31].

### 2.5. Nucleotide Sequence Polymorphism Analysis

Malaysian PCV2 *cap* gene nucleotide sequences obtained in this study were further analyzed for selection pressure. Tajima’s D, Fu and Li’s D, and Fu and Li’s F statistical tests of neutrality were performed using the DnaSP v6.12.03 software program [32,33,34]. Statistical significance was set at *p* ≤ 0.05 for all three tests. Positive and negative selective pressures acting on codon of the *cap* gene sequences were estimated based on calculated difference between non-synonymous (dN) and synonymous (dS) substitution rates per codon. Single-likelihood ancestor counting (SLAC), fixed-effects likelihood (FEL), fast, unconstrained Bayesian approximation (FUBAR) and mixed effects model of evolution (MEME) selection pressure inferring methods were run in the DataMonkey web server (http://www.datamonkey.org/, accessed on 2 May 2022) [35,36,37,38]. To infer dN and dS rates, FUBAR uses the Bayesian approach; FEL and MEME utilize the ML approach; while SLAC incorporates additional counting approaches. FEL, SLAC and FUBAR detects both positive and negative selection, but MEME focuses in detecting aa sites evolving under positive selection. For the FUBAR method, positively selected sites were identified when α < β at posterior probability (pp) > 0.9, where α represents the mean posterior synonymous substitution rate at a site and β represents the mean posterior non-synonymous substitution rate at a site. Negatively selected sites were identified when α > β at pp > 0.9. For the FEL method, positively selected sites were identified when β > α at *p* < 0.05, where α represents a synonymous substitution rate at a site and β represents the non-synonymous substitution rate at a site. Negatively selected sites were identified when β < α at *p* < 0.05. For the SLAC method, positively selected sites were identified when dN/dS > 1 at *p* < 0.05, where dS represents the inferred synonymous substitution rate at a site and dN represents the inferred non-synonymous substitution rate at a site. Negatively selected sites were identified when dN/dS < 1 at *p* < 0.05. For the MEME method, positively selected sites were identified when p+ > 0 at *p* < 0.05, where p+ represents the mixture distribution weight allocated to β+ and β+ represents the non-synonymous substitution rate at a site for positive selection. For each site where statistically significant selection pressure had been identified, statistical method consensus (n) across FUBAR, FEL, SLAC and MEME methods was recorded. Further, to evaluate aa residue diversity of PCV2 *cap* gene nucleotide sequences, Shannon entropy (Hx) values were calculated with BioEdit software v7.2.5 using the Shannon entropy formula: $-(\sum_{j=1}^{4}p_{ij}\log_{2}p_{ij})$ where *i; j* is equal to 1, 2, 3 and 4, corresponding to A, C, G and T nucleotides and *pij* being the proportion of nucleotide *j* in site *i.* Entropy plots of aa sequences of the *cap* genes were constructed to plot the diversity of aa residues at a given position [39,40]. In the context of diversity of aa residues at a given position, Hx value of entropy will present as 0.0 when only a single residue is present. As the Hx value increases, it would be more likely to observe different aa residues diversity at the same codon position. A Hx value of 4.322 indicates all 20 residues are equally represented. Aa with Hx > 2.0 are considered variable, while highly conserved aa would be expected to have Hx of < 1.0 [41]. Range, mean and standard error of mean (SEM) were calculated using previously described IBM SPSS Statistics [28].

### 2.6. Rates of Substitution

Estimations of the rate of substitution of PCV2 *cap* gene nucleotide sequences were calculated by running the Bayesian Markov chain Monte Carlo (MCMC) algorithm using BEAST v1.10.4 software [42]. Three independent MCMC runs were computed for the complete dataset of 241 PCV2 *cap* gene nucleotide sequences, under a relaxed molecular clock model, Hasegawa–Kishono–Yano (HKY) substitution model, gamma site heterogeneity model and the remaining default parameters in the prior’s panel. Bayesian Skygrid tree prior was selected to account for different population dynamics through time, set at 52 parameters and one time at last transition point [43,44]. The MCMC run was set with 2 × 10^8^ length of chain, with a posterior probability distribution of the long chains to be sampled every 1000 steps. Convergence to the same posterior distribution was assessed using Tracer v1.7.2, first by visual appraisal of the runs’ trace plot, followed by ensuring effective sampling size of greater than 200 after a 10% burn-in [45]. Estimations of rates of substitution were obtained from the three independent runs using LogCombiner software v1.10.4 (part of the BEAST v1.10.4 package), with 95% CI calculated. MCMC runs were then repeated as described above, on data subsets of PCV2a, PCV2b and PCV2d *cap* gene nucleotide sequences independently.

## 3. Results

### 3.1. Molecular Detection of PCV2

Out of the 79 domestic pigs sampled from 37 commercial swine farms in Peninsular Malaysia, 66 pigs were positive for PCV2 based on PCR detection, amounting to a molecular detection rate of 83.54%. At the farm level, 31 out of 37 farms (83.78%) were tested positive (Table 1). Notably, higher PCV2 molecular detection rates were observed in abattoir lung samples and wild boar samples: 18 out of 19 (94.74%) abattoir samples from clinically healthy finishers and all 28 (100%) wild boar lung samples were tested positive for PCV2 antigen.

Among the farm samples, PCV2 was detected across all production age groups as tabulated in Table 2.

Co-infection rate of PCV2 and PCV3 among the samples tested in this study was 28.77% (21/73). Relative risk of PCV2 and PCV3 co-infection was determined to be 1.25 (95% CI, *p*: 0.01); suggesting that PCV3 positive animals could be 25% more likely to be positive for PCV2 (Table 3). The Odds ratio was found to be statistically insignificant (OR: 6.46, 95% CI, *p*: 0.08).

### 3.2. Pairwise Sequence Comparison (PASC)

Pairwise distance estimation based on the p-distance nucleotide substitution model calculated across the 241 PCV2 *cap* gene nucleotide sequence dataset computed a p-distance value matrix ranging from 0.000 to 0.193. The sequences shared at least 80.7% of nucleotide identity similarities. Complete results of the pairwise distances are tabulated in Supplementary Tables S1 and S2. Excluding three strains that appeared to be outliers (PCV2/PG43I/UPM/MY007/OM524604, PCV2/WB1/UPM/MY052/OM524649 and PCV2/WB10/UPM/MY060/OM524657), the p-distance range narrowed to demonstrate that the Malaysian PCV2 *cap* gene nucleotide sequences shared at least 91.16% of nucleotide identity similarities. When sequences from domestic pigs and wild boars were compared independently, sequences from domestic pigs were more closely related to each other at 94% nucleotide identity similarities; compared to 88.46% when wild boar sequences were analyzed jointly.

**Figure 1:**
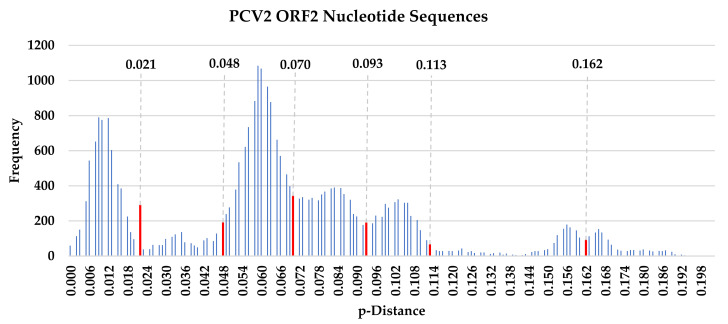
**PASC analysis of PCV2 cap gene nucleotide sequences.** Pairwise sequence comparison for PCV2 cap gene nucleotide sequences were calculated based on pairwise p-distances within a 0.01 p-distance interval. PASC distance thresholds were previously used to define PCV2a and PCV2b genotypes. Seven distance thresholds ranging from 0.021 to 0.162 were identified in this multimodal curve, denoted by red lines.

The pairwise sequence comparison (PASC) analysis based on PCV2 cap gene sequences resulted in a multimodal curve with several distance thresholds ranging from 0.021 to 0.162 (Figure 1). All the threshold values observed in this analysis did not match the previously proposed cut-off values of 0.035, 0.068 and 0.09 [31,46]. Further, only seven threshold values could be identified in this PASC graph. Numerous frequency bar clusters were too low for clear demarcation, leaving two ambiguous threshold points between 0.113–0.162 and two points between 0.162–0.201. This PASC analysis could not distinctly classify all currently known eight genotypes of PCV2.

### 3.3. Phylogenetic Analysis and Genotyping

Seventy-nine PCV2 *cap* gene nucleotide sequences were sequenced successfully in this study; 51 sequences from domestic pigs and 28 sequences from wild boars. The Malaysian PCV2 *cap* gene sequences reported in this study have been deposited at GenBank (http://www.ncbi.nlm.nih.gov, accessed 14 March 2022) under accession numbers OM524598–OM524676. NCBI Nucleotide-BLAST^®^ analysis results confirmed the identity of the 79 nucleotide sequences as PCV2, sharing over 99% similarity with at least 100 reference PCV2 *cap* gene nucleotide sequences recorded in GenBank. All but one of the Malaysian *cap* gene nucleotide sequences are 701 bp in length, encoding a 233 aa Cap protein; the exception being sequence OM524649 which is 702 bp long.

**Figure 2:**
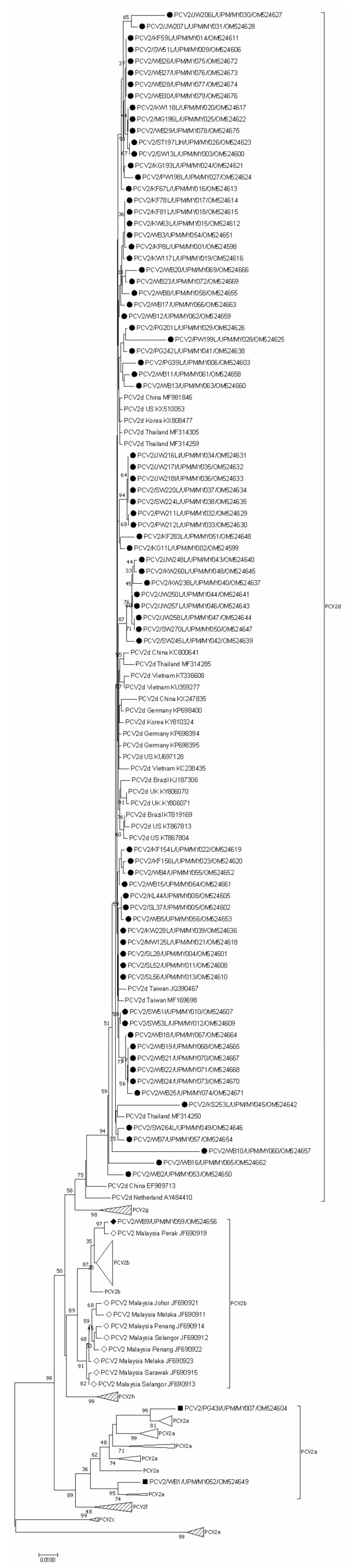
**Condensed phylogenetic analysis of PCV2 cap gene nucleotide sequences.** Seventy-nine Malaysian PCV2 cap gene nucleotide sequences (■ PCV2a, ◆ PCV2b, ● PCV2d) were compared with 162 other GenBank reference sequences from different countries and different genotype clades including previously published Malaysian sequences (denoted ◇). PCV2a and PCV2b clades were partially condensed, showing only Malaysian sequences. PCV2c, PCV2e, PCV2f, PCV2g and PCV2h clades were entirely condensed. GenBank accession numbers, origin country and genotype are as indicated. Malaysian PCV2 cap gene sequences were additionally labelled with sequence ID. The tree was constructed using NJ method, p-distance nucleotide substitution model with 1000 bootstrap replicates. The scale bar indicates branch length measured in number of substitutions per site.

A neighbor-joining (NJ) phylogenetic tree was constructed to analyze the 79 Malaysian PCV2 *cap* gene nucleotide sequences in relation to 162 GenBank reference sequences from different countries and different genotype clades. The complete and condensed resulting phylogenetic trees were identified as Supplementary Figure S1 and Figure 2, respectively. Wild boar *cap* gene nucleotide sequences were indicated with “WB” suffix. All 241 *cap* gene nucleotide sequences were grouped in genotype clades PCV2a through PCV2h. The resulting clustering matches the PCV2 genotype classification obtained by phylogenetic analysis generated based on a collection of strains representative of the proposed PCV2 genotypes [24].

**Figure 3:**
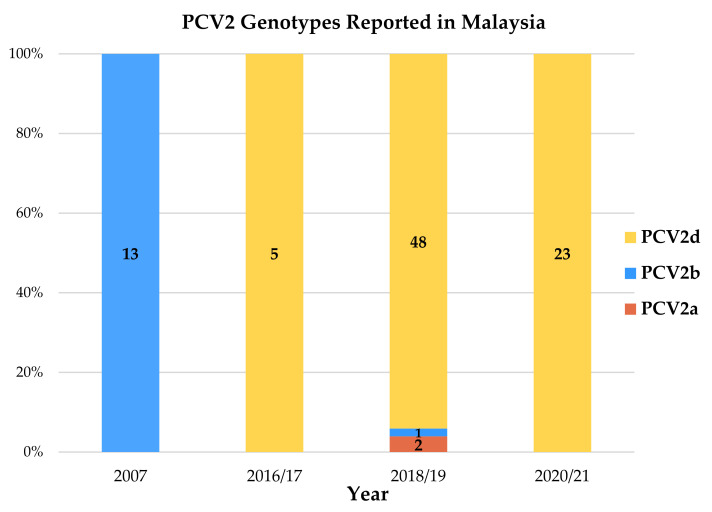
**Chronology of PCV2 genotypes reported in Malaysia.** PCV2 genotypes reported in Malaysia since year 2007 were tabulated chronologically to illustrate the trend of PCV2d genotype predominance. Figures within the bars represent total number of Malaysian PCV2 *cap* gene sequences.

The most apparent observation was that a 96.2% majority of the latest Malaysian sequences (76/79 sequences) were categorized as PCV2d. Among the domestic pig PCV2 strains, 98.04% (50/51) were classified as genotype PCV2d. As for the wild boar PCV2 strains, 92.86% (26/28) belonged to genotype PCV2d. Genotypes PCV2a and PCV2b were also identified in this study. The sole PCV2b sequence was identified from a wild boar sample (WB9/MY059/OM524656), sharing a clade with a Malaysian PCV2 strain dated back in year 2007 (JF690919), originating from the northern region. Two PCV2a sequences were identified, one strain collected from a domestic pig (PG43I/MY007/OM524604) and the other strain originating from a wild boar (WB1/MY052/OM524649). These phylogenetic-based clusterings were tallied with the pairwise distance analysis, which reaffirmed that the Malaysian PCV2 *cap* gene nucleotide sequences reported in this study shared more similarities with PCV2d reference strains in GenBank deposit. The sole Malaysian PCV2b sequence (WB9/MY059/OM524656) and the only two Malaysian PCV2a sequences (PG43I/MY007/OM524604 and WB1/MY052/OM524649) also turned out to be have further pairwise distances from all other Malaysian PCV2 strains. The PCV2 genotypes reported in Malaysia since year 2007, including the report from Jaganathan et al. (2011), were tabulated chronologically in Figure 3 [25].

When the genotypes of 79 Malaysian PCV2 cap gene nucleotide sequences were tallied with the genotype specific marker nucleotide system proposed by Franzo et al. (2015), Malaysian PCV2a and PCV2b strains fit completely into the system classification [31]. For Malaysian PCV2d strains, 88.16% (67/76) strains completely match the marker positions. The nine strains with dissimilar nucleotides at the marker positions were listed in Table 4.

Although wild boar sequences tend to form distinct clades, their clades and several individual strains could be found interspersed within clades of domestic pig sequences. Some clades between domestic pigs and wild boar sequences were supported by the substantial bootstrap of 33% to 97%. Generally, the Malaysian PCV2 *cap* gene nucleotide sequences tend to form large clusters according to their year of origin. This pattern was especially evident in the clade of year 2016/2017 sequences; and the large cluster containing the majority of year 2020/2021 sequences. As expected, sequences collected from a same farm at one single timepoint would be grouped in one clade, such as sequences JW216LI/MY034/OM524631, JW217I/MY035/OM524632, JW218I/MY036/OM524633 from Farm S5; SW220L/MY037/OM524634, SW224L/MY038/OM524635 from Farm C8/C9; SW51I/MY010/OM524607, SW53L/MY012/OM524609 from Farm C1; and JW257L/MY046/OM524643, JW258L/MY047/OM524644 from Farm S2. However, when the sampling interval spanned over months to years, sequences originating from one farm may no longer share a close phylogenetic relationship. For instance, sequences KG11L/MY002/OM524599, KW63L/MY015/OM524612, KF67L/MY016/OM524613, KF78L/MY017/OM524614, KF81L/MY018/OM524615 and KF283L/MY051/OM524648 collected from Farm N3 over year 2018/2019 to year 2020/2021, were dispersed from each other even though they were still within a bigger cluster. In February 2019, the PCV2a genotype (PG43I/MY007/OM524604) was detected in Farm N4. Later in December 2019 and July 2020, strains from the same farm (PG201L/MY029/OM524626 and PW212L/MY033/OM524630) were identified as genotype PCV2d.

A unique phylogenetic pattern was noted for two neighboring farms: Farm N4 and N7. PCV2 strains of the two farms appeared to evolve phylogenetically in tandem. Sequence PW212L/MY033/OM524630 (Farm N4) and sequence PW211L/MY032/OM524629 (Farm N7) collected in year 2020/2021 were observed to be closely related; just as sequence PG201L/MY029/OM524626 (Farm N4) and sequence PW199L/MY028/OM524625 (Farm N7) collected back in year 2018/2019 were found with a closer phylogenetic relationship. Furthermore, the co-existence of two different PCV2 strains within one individual pig was noted when sequences PCV2/SW51L/UPM/MY009/OM524606 and PCV2/SW51I/UPM/MY010/OM524607 obtained from lung and inguinal lymph node, respectively, of the same pig were found to be genetically and phylogenetically distinct.

### 3.4. Nucleotide Sequence Polymorphism Analysis

To determine positive and/or negative selections in the PCV2 *cap* gene nucleotide sequences, neutrality test values, dN–dS values and Hx values of the *cap* gene were evaluated. The sequences were analyzed separately according to their genotype: PCV2a, PCV2b or PCV2d. Results from the statistical tests of neutrality, ran on the PCV2 *cap* gene nucleotide sequences, were summarized in Table 5. All three tests demonstrated unanimous negative neutrality values, of which analysis values for PCV2b and PCV2d genotypes demonstrated statistical significance.

### 3.5. Nucleotide Sequence Polymorphism Analysis

Further, positive and negative selective pressures acting on codons of PCV2 cap gene nucleotide sequences were determined based on a calculated difference between non-synonymous (dN) and synonymous (dS) substitution rates per codon. Summary of single-likelihood ancestor counting (SLAC), fixed-effects likelihood (FEL), fast, unconstrained Bayesian approximation (FUBAR) and mixed effects model of evolution (MEME) selection pressure inference results were tabulated in Table 6. Sites positively and negatively selected and identified under the various selection pressure inferring methods were listed in Supplementary Table S3 with details of their respective selective criteria. At the statistical significance thresholds applied in this study, a higher number of negative selections was observed across all PCV2 genotypes. Up to 13.73% (32/233) of codons were identified to be under negative selection, contrasting to only 1.72% (4/233)–3.86% (9/233) of codons identified to be influenced by positive selection. The proportion of negatively selected codons reaching consensus across all selection pressure inferring methods was also higher in the negative selection group, at 28.13% (9/32)–43.75% (14/32), compared to the 14.29% (1/7) in the positive selection group. Among the four positive selection pressure inferring methods used, descending detection rates were observed from the MEME, FUBAR, FEL to SLAC method. A similar detection rate pattern was observed across FUBAR, FEL to SLAC methods for negative selection.

**Figure 4:**
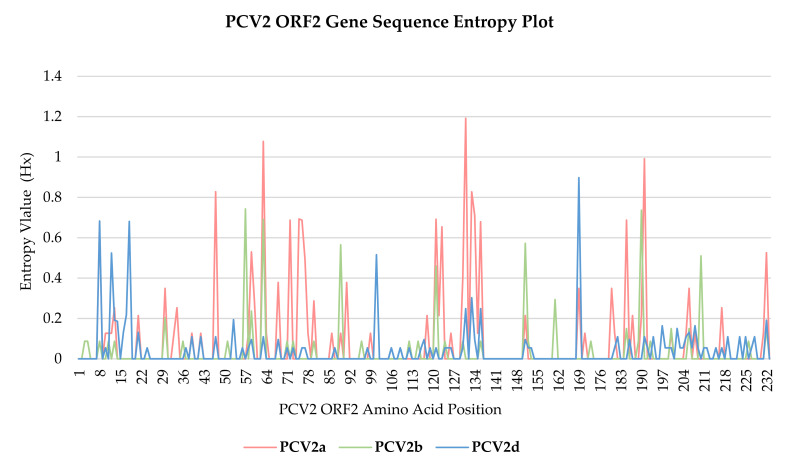
Entropy plot of PCV2 ORF2 gene sequences. Shannon entropy analysis were carried out separately for each genotype PCV2a, PCV2b and PCV2d; and Hx value for each aa position was plotted.

Shannon entropy value (Hx) for each aa position of PCV2 cap gene codon was plotted in a line chart (Figure 4), with the analysis summarized in Table 7. With the exception of aa position 63 and 131 of genotype PCV2a, where the Hx values were 1.078 and 1.192, respectively, all the recorded Hx values were well below 1.0. Comparatively among genotypes, the PCV2d cap gene nucleotide sequences have the most aa sites with entropy values of >0.0, at 31.76% (74/233), with values ranging from 0.055 to 0.8977.

### 3.6. Rates of Substitution

Estimations of the rate of substitution of PCV2 *cap* gene nucleotide sequence dataset generated from the Bayesian Markov chain Monte Carlo (MCMC) algorithm were detailed in Table 8. For all the replicate runs in the three independent MCMC analysis, all parameters and densities of interest were found to converge to the same posterior distribution of a 10% burn-in, with large effective sample size (ESS) values well exceeding 200.

Overall, when all three genotypes PCV2a, PCV2b and PCV2d were analyzed collectively, the substitution rate for the PCV2 cap gene was estimated to be 1.374 *×* 10^−3^ substitutions per site per year (ssy). When fractionated according to genotypes, the estimated substitution rate of PCV2d genotype was the highest at 2.111 *×* 10^−3^ ssy; compared to that of 4.746 *×* 10^−4^ ssy and 6.571 *×* 10^−4^ ssy observed in PCV2a and PCV2b genotypes, respectively.

## 4. Discussion

The first case of porcine circovirus 2 (PCV2) in Malaysia was reported by the Malaysian Veterinary Research Institute. The said PCV2 DNA was identified using a PCR-restriction fragment length polymorphism (PCR-RFLP) assay in a respiratory-related clinical case [47]. Later, PCV2 was reported in the first-described post-weaning multisystemic wasting syndrome (PMWS) clinical case in Malaysia, with a PCR molecular detection rate of 83.33% (10/12 organ samples) [48]. The last published update of Malaysian PCV2 reported a PCR molecular detection rate of 88.10% (37/42 farms) [25]. This present study reported a similar level of prevalence: 83.78% (31/37) at farm level and 83.54% (66/79) in the sampled domestic pig population. The southern region of Malaysia demonstrated the lowest molecular detection rate, possibly due to farther farm-to-farm distances, which contribute to stronger biosecurity control.

Previously, the presence of PCV2 strains in Malaysia was only reported in the clinically ill domestic pig population. This study reported a relatively high molecular detection rate of 94.74% (18/19) in abattoir samples originating from clinically healthy finishers. PCV2 was also detected in clinically ill pigs from the same farms that supplied to the said abattoirs. It is known that PCV2 can be present in both clinically healthy and ill pigs, in pigs with or without PMWS presentation. This observation suggested the need for additional infectious and non-infectious factors to trigger PCV2 replication up to an extent of overwhelming the immune system [49,50,51,52]. Although the implication of detecting PCV2 in healthy animals in this study still warrants further investigation, the potential PCV2 transmission amongst swine herds through apparently healthy animals should not be ignored [53]. Detection across all production age groups from fetus, piglet, weaner, grower to sow at 80.36% (45/56) to 100% (7/7) was reported in this study, reiterating that PCV2 infection can occur across all pig production age groups [54,55]. PCV-associated disease (PCVAD) manifests as different clinical syndromes, and also as subclinical infection without a conspicuous clinical sign has been demonstrated to be associated with PCV2 [52,56].

Further, this is the first study to confirm the circulation of PCV2 in the wild boar population roaming Peninsular Malaysia. PCV2 antigen was detected in all (28/28) of the tested wild boar organ samples. Serological and direct detection of PCV2 antigens in wild boars have been reported in various countries including Belgium, Brazil, Croatia, Czech Republic, Germany, Greece, Hungary, Italy, Korea, Poland, Romania, Serbia and Uruguay [31,57,58,59,60,61,62,63,64,65,66,67,68,69]. A seroprevalence of 20–48% have been reported in the European wild boar population, suggesting a high circulation rate of PCV2 [70]. All 28 Malaysian wild boar PCV2 *cap* gene nucleotide sequences in this study shared at least 80.7% of nucleotide identity similarities with Malaysia domestic pig PCV2 *cap* gene sequences. Reports from Greece, Hungary and Germany also found wild boar PCV2 isolates to be identical to those from domestic pigs, even when the sampling regions were distant [59,60,62]. In this study, clades of Malaysian wild boar sequence were observed to be interspersed among clades of domestic pig sequences, with several clades between domestic pigs and wild boar sequences supported by a substantial bootstrap of 33% to 97%. This could suggest a possible epidemiological link between wild boar and domestic pig populations [60,71]. Pig farms in Malaysia are usually located in remote areas, in the vicinity of forests and oil palm plantations. Considering the increasing population and migratory behavior of wild boars, contact between domestic pigs and wild boars and the resulting two-way pathogen transmission is highly possible [72,73]. Direct contact between the domestic pigs and wild boars, or indirect contact through fomites such as leftover feed, are both possible. Moreover, through Thailand, Peninsular Malaysia is linked to neighboring countries including Vietnam and China, which are intersected by one of the many possible wild boar migratory routes [73]. It was noted that the wild boar sequences clustered with domestic pig sequences from every state except for Johor. The farms sampled in Johor have farther farm-to-farm distances and stronger biosecurity control including proper perimeter fencing, which could minimize contact between wild boar and domestic pigs. However, the sample size from the region needs to be increased to be more conclusive.

Incidences of PMWS have also been reported in wild boars with clinical presentation and microscopic lesions similar to PMWS in domestic pigs [58,62,63]. Recently, PCV2 was detected in samples of pregnant wild boar and their fetuses, suggesting that PCV2 may have similar tropism for fetal tissues and thus is also involved in PCV-RD just as in domestic sows [74]. A high seroprevalence of PCV2 and widespread geographical distribution pattern may suggest an endemic status of PCV2 in the wild boar population [58]. Histopathologic lesions consistent with typical PMWS had been reported in wild boars [58,75,76]. More recently, a case close to fulfilling PMWS’s definition has been published: clinical signs of wasting and dyspnea were observed and PCV2 antigens were detected in lung, heart, intestine and lymph nodes samples. Moreover, the wild boar in question was aged less than six to eight months old, nearer to the PMWS susceptible age group in cases of domestic pigs [56,62]. Wild boars may potentially act as a reservoir of PCV2, given that (1) there are a large number of wild boars and they may come in come in contact with confined domestic pigs; (2) PCV2 have been shown to infect wild boar and could cause PMWS; (3) PCV2 could transmit from wild boars to domestic pigs [70,77,78,79]. It has been suggested that wild boars are a potential reservoir of viruses infectious to domestic pigs, and possibly vice versa from domestic pigs to wild boars. The reported virus transmission between these two groups include African swine fever, classical swine fever virus, Aujeszky’s disease virus and bovine viral diarrhea virus [80,81,82,83,84]. Considering the significant impact of PCV2 disease in domestic pig population, the direction of PCV2 transmission between domestic pigs and wild boars, as well as the similarity of pathogenesis and clinical manifestation of PCV2 infection between the two groups, should be further investigated. At the time being, avoiding close contact between wild boars and domestic pigs should be highlighted as an integral part of the PCV2 disease control program in farms. With the recent ASF outbreaks in several high domestic pig density areas, this biosecurity measure is presently especially critical in the Malaysian domestic pig farming scenario. Sightings of wild boars near pig farms are not uncommon; thus, contact between wild boars and domestic pigs is certainly plausible and must be managed.

This study also reports co-detection of PCV2 and PCV3, where a relative risk analysis found PCV3-positive pigs to be 25% more likely to be positive for PCV2. Porcine circovirus 3 (PCV3) and porcine circovirus 4 (PCV4) are two newly emerging viruses in the swine industry, first reported in 2016 and 2019 [6,7]. Reports of co-infection of PCV2 with these two relatively new PCV species have surfaced, with the clinical implication of their co-existence in the swine host still being debated [85,86,87,88]. PCV3-positive clinical samples were frequently found to be co-infected with PCV2 with co-infection rates of 27.6% to 69.74%, suggesting the common occurrence of mixed PCV2 and PCV3 infection [86,89,90]. In view that both PCV2 and PCV3 predominantly infect macrophages of lymphoid tissues, co-infection of PCV2 and PCV3 could have clinical implication worth investigating further [6,91].

The genomic variability of Malaysian PCV2 strains were compared by having their 701–702 bp long *cap* gene nucleotide sequences phylogenetically analyzed, as the cap gene is the most variable region spanning the entire PCV2 genome sequence [11,19]. It has been established that PCV2 displays very high substitution rates, which are higher than expected for a DNA virus and instead akin to RNA viruses [92]. For the PCV2 *cap* gene nucleotide sequences in this study, the estimated substitution rates for PCV2a, PCV2b and PCV2d genotypes were 4.746 × 10^−4^, 6.571 × 10^−4^ and 2.111 × 10^−3^ substitutions/site/year (ssy), respectively. When all the sequences were analyzed together, the overall estimated rate was 1.102 × 10^−3^ ssy. High substitution rates as such, within the order of magnitude of 10^−3^–10^−4^, were in agreement with previous reports [22,31,93,94]. Furthermore, co-existence of two different PCV2 strains within one individual pig was observed, when PCV2 strains obtained from lung and inguinal lymph node, respectively (PCV2/SW51L/UPM/MY009/OM524606 and PCV2/SW51I/UPM/MY010/OM524607), originating from the same pig, were found to be non-identical genetically, sharing 99.43% nucleotide identity similarity and belonging to rather distant phylogenetic clades. The circulation of different PCV2 strains in a farm, or concurrent infection of different strains in the same pig, were suggested as the potential for viral recombination, through recombination of two parental strains with various patterns and crossover regions [95,96,97,98]. Strain clustering according to the year of origin was also a pattern observed in the phylogenetic analysis of this study. The evident clustering of year 2016/2017 clade and year 2020/2021 clade could suggest the mutation of PCV2 strains that occurs over time. As reported in the results section, when longitudinal sampling was carried out in the same farm, the sequences demonstrated a further phylogenetic relationship when the sampling interval spanned longer over months to years, again proffering speculation of the PCV2 strain mutation with time. High substitution rates can represent a substrate for selective pressures to act [94]. The unanimous negative values of all three neutrality tests signify an excess of low frequency polymorphisms relative to expectation, an effect resulting from either purifying the selection or expansion of population size [32,33]. Considering the general negative trend of dN–dS values, as shown in Table 7 and Supplementary Table S3, it was suggested that *cap* genes of PCV2 may be heavily influenced by negative selection pressure. In this study, *cap* gene codons indicated a higher negative selection of 13.73% (32/233) compared to positive selection of 1.72–3.86% (4–9/233) at the significance threshold used in this study, translating as a strong negative selection acting over the *cap* gene of PCV2. In the context of Shannon entropy values (Hx), a large majority of the recorded Hx values were well below 1.0, supporting the conjecture of the PCV2 *cap* gene being under strong purifying selection processes [11].

The pairwise sequence comparison (PASC) analysis based on PCV2 *cap* gene nucleotide sequences resulted a multimodal curve with several distance thresholds, ranging from 0.021 to 0.162. This methodology could not distinctly classify all currently known eight genotypes of PCV2 [24]. A neighbor-joining phylogenetic tree based on pairwise p-distance with 1000 bootstrap replicates divided the PCV2 strains into eight clusters, matching the previously published genotype classification [24]. Most importantly, the most apparent observation in this study was a genotype shift from genotype PCV2b to PCV2d. In this study, 96.2% of the majority of the latest Malaysian sequences (76/79 sequences) were identified to be genotype PCV2d; a stark contrast to the previous Malaysian study conducted ten years ago, which reported only PCV2b strains [25]. However, the limitation of sample size in the aforementioned study needs to be taken into account. Nevertheless, the clear-cut predominance of genotype PCV2d revealed in this study warrants our attention. This observation of genotype shift matches the well-described PCV2 epidemiological dynamics, characteristically beginning with an initial rise of genotype PCV2a in the 90s, which was rapidly superseded by the wave of PCV2b about ten years later [46,99,100,101,102,103]. The rise of PCV2a and the subsequent shift to PCV2d broadly corresponds to incidences of PCVAD and to the significant increase in frequency and severity of clinical PCVAD outbreaks, respectively [99,100,101,104]. The results of this study matched the current occurrence of a second major PCV2 genotype shift, where the emergence and spread of PCV2d strains is set in motion to replace the PCV2b genotype [105]. Both natural infection and vaccine-induced responses were considered, given the ubiquitous nature of PCV2 and the widespread awareness of the PCV2 vaccination practice. In Malaysia, PCV2 vaccination is a commonly accepted practice. Currently available commercial PCV2 vaccines, including Circovac^®^ (Ceva), Ingelvac CircoFLEX^®^ (Boehringer Ingelheim), Fostera^®^ PCV(Zoetis) and Porcilis^®^ (MSD), are all inactivated vaccines based on the PCV2a genotype. During prolonged circulation in a highly immune population, PCV2 continuously mutates its antigenic features to evade from immune responses [94]. The majority of Malaysian PCV2d strains were sequestered into seven clusters of their own. Some of the clusters shared clades with PCV2d strains from numerous countries, including Canada, Denmark, France, Sweden, South Korea and U.S., with whom Malaysia has trade relationship involving breeder importation as early as back in year 2000 [106]. Hence, it may be speculated that the phylogenetic relatedness might be a result of live breeder and semen stock movement. Phylogenetic relationship was also shared among Malaysian PCV2 strains with Thailand, Vietnam and China, possibly due to geographical factor and trade activities involving porcine products [106].

## 5. Conclusions

This study reported PCV2 molecular detection rates of 83.78% and 83.54% at a farm and sample population level, respectively, close to the last published update on Malaysian PCV2 status. A high molecular detection rate of 94.74% was reported in clinically healthy finishers. Given these high detection rates at farm and abattoir levels, it could be beneficial to investigate both subclinical and clinical PCVAD more thoroughly at the farms. Further, this study also confirmed the circulation of PCV2 in the wild boar population roaming Peninsular Malaysia. Malaysian wild boar PCV2 sequences were found to be interspersed among clades of domestic pig sequences, sharing at least 80.7% of nucleotide identity similarities. Although the pathogenesis and clinical manifestation of PCV2 in wild boars have yet to be elucidated, the potential of wild boars as a PCV2 reservoir should be considered. Most notably, an apparent genotype shift away from the sole PCV2b genotype reported ten years ago in Malaysia was observed. This study revealed that the 96.2% majority of the latest Malaysian sequences (76/79 sequences) were identified as genotype PCV2d. Taken together, findings of continual high molecular detection rates in farms; PCV2 antigen detection in clinically healthy abattoir samples and in wild boar population; and most importantly, a new wave of genotype shift from PCV2b to PCV2d—warrant further research on the Malaysian PCV2 and PCVAD situation to facilitate direction of control and management applicable to the local situation.

## Figures and Tables

**Table 1:** **PCV2 PCR positive rate reported in this study.** Samples consisted of organ samples collected from domestic pig farms, abattoir and wild boar population of Peninsular Malaysia.

PCV2 PCR Detection Rate	Domestic Pig				Abattoir	Wild Boar
	Northern Malaysia	Central Malaysia	Southern Malaysia	Overall		
% PCR Positive (Animals)	93.10% (27/29)	82.76% (24/29)	71.43% (15/21)	83.54% (66/79)	94.74% (18/19)	100.00% (28/28)
% PCR Positive (Farms)	92.86% (13/14)	85.71% (12/14)	66.67% (6/9)	83.78% (31/37)	-	-

**Table 2:** **Distribution of PCV2 positive samples across production age groups.** Molecular detection status of PCV2 was tabulated for each production age group from fetus to sow.

Production Age Group	% PCR Positive (Number of Positive Animals/Tested Animal)
Fetus	100% (7/7)
Piglet	100% (2/2)
Weaner	80.36% (45/56)
Grower	83.33% (10/12)
Sow	100% (2/2)

**Table 3:** **Distribution of PCV2 and PCV3 co-infection molecular detection status.** The relative risk (RR) and Odds ratio (OR) of PCV2 co-infection with PCV3 were computed based on the molecular detection status tabulated above.

Detection Status of Tested Pig (Number of Individuals)	PCV2 Positive	PCV2 Negative
PCV3 Positive	21	1
PCV3 Negative	39	12

**Table 4:** **Malaysian PCV2d cap gene nucleotide strains agreement with marker nucleotide positions.** Malaysian PCV2a and PCV2b strains match completely into Franzo et al. (2015) system classification. Nine PCV2d strains that did not fit into the system were tabulated above together with overall agreement %.

	Marker Nucleotide Position	157 A	162 T	513 *C*	585 T	643 A
Malaysian PCV2d Sequences						
KL44/OM524605		A	T	A	T	A
SL37/OM524602		A	T	A	T	A
PG201L/OM524626		A	T	A	T	A
PG242L/OM524638		A	T	A	T	A
PG39L/OM524603		A	T	A	T	A
PW199L/OM524625		A	T	A	T	A
WB2/OM524650		T	A	C	T	A
WB5/OM524653		A	T	A	T	A
WB10/OM524657		T	T	C	T	A
**% Sequences not matching marker nucleotides**		2.63% (2/76)	1.32% (1/76)	9.21% (7/76)	0.00%	0.00%

**Table 5:** **Results tabulation of PCV2cap gene nucleotide sequences statistical tests of neutrality.** PCV2 *cap* gene nucleotide sequences were analyzed separately according to their genotype to obtain their neutrality test values, which determine positive and/or negative selection pressure.

PCV2 Genotype	Tests of Neutrality					
	Tajima’s D		Fu and Li’s D		Fu and Li’s F	
	Test Statistic	Statistical Significance, *p*-Value	Test Statistic	Statistical Significance, *p*-Value	Test Statistic	Statistical Significance, *p*-Value
PCV2a	−1.2028	>0.10	−2.2422	0.10 > *p* > 0.05	−2.2331	0.10 > *p* > 0.05
PCV2b	−2.1293	<0.05	−3.3600	<0.05	−3.4549	<0.02
PCV2d	−2.5171	<0.001	−5.8800	<0.02	−5.2945	<0.02

**Table 6:** **Selection pressures acting on codons of PCV2 cap gene nucleotide sequences.** Proportion of codons under statistically significant positive and negative selective pressure were identified. Selection pressure inferring methods applied were FUBAR, FEL and SLAC for both positive and negative selection; with an additional method MEME for positive pressure. Statistical significance was set at pp > 0.9 for FUBAR and *p* < 0.05 for FEL, SLAC and MEME.

PCV2 Genotype	Positive Selection									Negative Selection						
	Total Positively Selected Codon	Selection Pressure Inference Methods				Statistical Consensus across Methods				Total Negatively Selected Codons	Selection Pressure Inference Methods			Statistical Consensus across Methods		
		FUBAR	FEL	SLAC	MEME	n = 1	n = 2	n = 3	n = 4		FUBAR	FEL	SLAC	n = 1	n = 2	n = 3
PCV2a	3.86% (9/233)	3	1	0	7	8	0	1	0	13.73% (32/233)	29	31	14	4	14	14
PCV2b	1.72% (4/233)	3	0	0	1	4	0	0	0	13.73% (32/233)	30	22	9	12	11	9
PCV2d	3.00% (7/233)	3	1	1	6	5	1	0	1	13.30% (31/233)	27	26	9	9	13	9

**Table 7:** **Shannon entropy values for PCV2 cap gene nucleotide sequences.** Hx values computed for each aa position of the gene nucleotide sequences were summarized separately for each genotype, PCV2a, PCV2b and PCV2d.

PCV2 Genotype	ORF2 aa Site with Hx Values > 0.0	Lowest Hx Value	Highest Hx Value	Standard Deviation, σ
PCV2a (36)	23.61% (55/233)	0.127	1.192	0.208
PCV2b (58)	15.02% (35/233)	0.087	0.743	0.111
PCV2d (103)	31.76% (74/233)	0.055	0.898	0.108

**Table 8:** **Estimations of rate of substitution for PCV2 cap gene nucleotide sequences.** Estimations of the rates of substitution were generated from Bayesian MCMC algorithm. For all replicate runs of the three independent MCMC analysis, the results were found to fit burn-in and ESS validity criteria.

PCV2 Genotype	Mean Rate of Substitution (Substitution per Site per Year)	95% Highest Posterior Density (HPD)	Effective Sample Size (ESS)
PCV2a	4.746 × 10^−4^	8.463 × 10^−4^	246,638
PCV2b	6.571 × 10^−4^	1.024 × 10^−3^	255,628
PCV2d	2.111 × 10^−3^	2.925 × 10^−3^	68,174
Combined analysis for all genotypes	1.102 × 10^−3^	1.374 × 10^−3^	82,401

## Data Availability

The Malaysian PCV2 *cap* gene sequences reported in this study have been deposited at GenBank (http://www.ncbi.nlm.nih.gov) under accession numbers OM524598–OM524676.

## Supplementary Materials

The following supporting information can be downloaded at: https://www.mdpi.com/article/10.3390/ani12141849/s1, Supplementary Table S1. Pairwise distance analysis of *cap* gene nucleotide sequences of PCV2, shown as p-distance values. Seventy-nine Malaysian PCV2 strains and 162 PCV2 GenBank reference strains were compared. The analysis was run using the pairwise distance method and p-distance method evaluated with 1000 bootstrap replicates. Supplementary Table S2. Pairwise distance analysis of *cap* gene nucleotide sequences of PCV2, shown as percentage nucleotide identity similarity. Seventy-nine Malaysian PCV2 strains and 162 PCV2 GenBank reference strains were compared. The analysis was run using pairwise distance method and p-distance method evaluated with 1000 bootstrap replicates, as described in Supplementary Table S1. Percentage nucleotide identities of ≤95.00% are indicated in red boxes. Supplementary Figure S1. Complete phylogenetic analysis of PCV2 *cap* gene nucleotide sequences. Seventy-nine Malaysian PCV2 *cap* gene nucleotide sequences (■ PCV2a, ◆ PCV2b, ● PCV2d) were compared with 162 other GenBank reference sequences from different countries and different genotype clades, including previously published Malaysian sequences (denoted ◇). GenBank accession numbers, origin country and genotype are as indicated. Malaysian PCV2 *cap* gene sequences were additionally labelled with sequence ID. The tree was constructed using NJ method, p-distance nucleotide substitution model with 1000 bootstrap replicates. The scale bar indicates branch length measured in number of substitutions per site. Supplementary Table S3. Selection pressures acting on codons of PCV2 *cap* gene nucleotide sequences. Each codon under statistically significant positive and negative selective pressure were listed with their respective statistical details. Selection pressure inferring methods applied were FUBAR, FEL and SLAC for both positive and negative selection; with an additional method MEME for positive pressure. Statistical significance was set at pp > 0.9 for FUBAR and *p* < 0.05 for FEL, SLAC and MEME.
