# Supplementary material for: Genotype Shift of Malaysian Porcine Circovirus 2 (PCV2) from PCV2b to PCV2d within a Decade
Source: Animals (Basel). 2022 Jul 21;12(14):1849. doi: 10.3390/ani12141849 (PMC9311952; doi:10.3390/ani12141849)
Supplement: Supplementary file 1 [file animals-12-01849-s001.zip › Supplementary Table S3 [Genotype Shift of Malaysian PCV2].pdf]

Supplementary Table S3. Selection pressures acting on codons of PCV2 *cap* gene nucleotide sequences. Each codon under statistically significant positive and negative selective pressure were listed with their respective statistical details. Selection pressure inferring methods applied were FUBAR, FEL and SLAC for both positive and negative selection; with an additional method MEME for positive pressure. Statistical significance were set at pp > 0.9 for FUBAR and p < 0.05 for FEL, SLAC and MEME.

Positive Selection

PCV2 Genotype	PCV2a											PCV2b											PCV2d										
	FUBAR			FEL			SLAC			MEME		FUBAR			FEL			SLAC			MEME		FUBAR			FEL			SLAC			MEME	
Identified Sites	α	β	α < β, at pp > 0.9	α	β	β > α, p < 0.05	dS	dN	dN/dS > 1, at p < 0.05	p+	p+ > 0, p < 0.05	α	β	α < β, at pp > 0.9	α	β	β > α, p < 0.05	dS	dN	dN/dS > 1, at p < 0.05	p+	p+ > 0, p < 0.05	α	β	α < β, at pp > 0.9	α	β	β > α, p < 0.05	dS	dN	dN/dS > 1, at p < 0.05	p+	p+ > 0, p < 0.05
8																							1.489	13.121	0.959								
12																							0.776	8.208	0.979							0.2	0
17																																0.18	0.04
57												0.995	11.629	0.972																			
59										0.11	0.02										0.03	0.01										0.01	0
63	0.888	6.846	0.969																														
68										0.11	0.04																					0.02	0.02
86										0.04	0.02																						
89												0.908	8.077	0.957																			
130										0.09	0																						
131																																0.01	0
169										0.02	0												0.87	13.582	0.994	0.764	5.284	0.0244	1.13231	7.67557	0.02023	0.5	0.01
190												0.752	5.239	0.919																			
191	0.683	6.657	0.987	0	3.113	0.029				0.5	0.04																						
231										0.02	0																						
232	1.216	5.183	0.911																														

Negative Selection

PCV2 Genotype	PCV2a											PCV2b											PCV2d										
	FUBAR			FEL			SLAC			MEME		FUBAR			FEL			SLAC			MEME		FUBAR			FEL			SLAC			MEME	
Identified Sites	α	β	α < β, at pp > 0.9	α	β	β > α, p < 0.05	dS	dN	dN/dS > 1, at p < 0.05	p+	p+ > 0, p < 0.05	α	β	α < β, at pp > 0.9	α	β	β > α, p < 0.05	dS	dN	dN/dS > 1, at p < 0.05	p+	p+ > 0, p < 0.05	α	β	α < β, at pp > 0.9	α	β	β > α, p < 0.05	dS	dN	dN/dS > 1, at p < 0.05	p+	p+ > 0, p < 0.05
9	6.54	0.323	0.991	3.775	0	0.0058	2.76	0	0.048259			11.923	0.478	0.999	3.763	0	0.0065	3.31	0	0.026582													
10																							7.42	0.61	0.983	4.133	0.315	0.0153					
11	10.355	0.772	0.995	5.334	0.534	0.0109	4.93	0.645	0.031497																								
15																							22.562	0.451	1	11.754	0	0.0001	6	0	0.001372		
16																							10.49	1.704	0.939								
21	8.837	0.664	0.951	5.03	0.399	0.0378																											
35	4.238	0.32	0.957	2.81	0	0.0166																											
37	4.325	0.321	0.957	2.81	0	0.0174						31.306	0.502	1	13.486	0	0	4.96	0	0.004302													
40																							5.24	0.286	0.991	3	0	0.0026	3.43785	0	0.024851		
43															2.605	0	0.0444									1.883	0	0.044					
49												17.479	0.543	0.998	7.721	0	0.0061	2.9	0	0.040893													
54																							4.07	0.288	0.967	2.81	0	0.0077					
62												11.696	0.471	0.985	5.357	0	0.0084						5.207	0.328	0.97	3.629	0	0.008					
64												5.487	0.474	0.946									2.136	0.352	0.909								
68												5.462	0.447	0.951																			
69																							2.119	0.325	0.919	1.353	0	0.0431					
70												9.069	0.437	0.922	7.204	0	0.0168																
74												7.821	0.491	0.902	6.046	0	0.038																
78																							3.907	0.296	0.922	4.865	0	0.0157					
79																										4.755	0	0.0258					
85																										1.905	0	0.0433					
91												5.441	0.454	0.949									5.093	0.316	0.994	2.518	0	0.005	4	0	0.012346		
99																							5.45	0.288	0.991	3.157	0	0.0024	3.466643	0	0.024676		
100				2.37	0	0.0471																	8.293	0.316	0.996	4.755	0	0.0012	5.237771	0	0.008633		
103	4.079	0.338	0.951	2.81	0	0.0208																	3.97	0.323	0.959	2.81	0	0.0133					
105												9.081	0.492	0.912	7.171	0	0.0321																
108	4.811	0.34	0.909	5.454	0	0.025																	6.962	0.335	0.932	8.808	0	0.0111					
110	5.77	0.315	0.988	3	0	0.0104	3	0	0.037037			9.949	0.499	0.993	3.061	0	0.0389	3	0	0.037037			2.686	0.375	0.918								
113																							7.695	0.329	0.995	4.414	0	0.0019	5.27213	0	0.008686		
115	25.558	0.361	1	20.205	0	0	8.35	0	0.00172																								
118	8.99	1.557	0.917																														
119	3.79	0.32	0.95	2.389	0	0.0212									2.614	0	0.0449																
125												5.898	0.536	0.943																			
127	7.284	0.311	0.981	6.701	0	0.0027	4.34	0	0.023551																								

136												9.777	1.307	0.914																			
140	7.161	0.321	0.993	4.191	0	0.005	3	0	0.037037																								
141	6.972	0.355	0.975	6.376	0	0.0066	4.17	0	0.037453																								
144												9.082	0.492	0.912	7.171	0	0.0303																
148				2.81	0	0.0447																											
149	5.677	0.318	0.987	3.054	0	0.0098	3	0	0.037037																								
150	5.532	0.337	0.966	3.805	0	0.0136																											
152	30.083	0.371	1	15.762	0	0	10.5	0	0.000392			23.039	1.103	0.997	7.385	0.591	0.0099	6.24	0.527	0.016245			9.871	0.652	0.99	5.352	0.359	0.0117	5.537488	0.447202	0.025332		
154												9.186	0.492	0.913	7.239	0	0.0316						7.519	0.364	0.927	9.407	0	0.0134					
159	7.759	0.338	0.994	4.503	0	0.0044						17.39	0.482	0.999	7.602	0	0.0016	2.95	0	0.039082			2.782	0.338	0.957	1.598	0	0.0331					
160												9.016	0.492	0.911	6.99	0	0.0306																
161	19.016	0.384	0.999	15.226	0	0.0002	7.63	0	0.002248			11.152	1.176	0.938												4.877	0	0.0254					
162	5.99	0.319	0.989	3.269	0	0.0084	3	0	0.037037			18.48	0.513	0.999	8.223	0	0.0027	3	0	0.037037													
163														0.569									2.239	0.376	0.903								
166	7.166	0.338	0.992	4.519	0	0.0047	3	0	0.037037			11.371	0.47	0.984	5.233	0	0.0087						5.161	0.328	0.97	3.671	0	0.008					
170	4.158	0.324	0.955	2.81	0	0.0184																											
171																							12.934	0.376	0.998	8.574	0	0.0006	4.391377	0	0.011809		
175	4.343	0.313	0.913	3.28	0	0.0347																											
177	7.414	0.32	0.981	6.828	0	0.003	4.3	0	0.024091			7.8	0.459	0.908	6.046	0	0.0257																
180																																	
181												11.198	0.466	0.984	7.186	0	0.0047	3.25	0	0.042171													
183	5.782	0.347	0.985	3.349	0	0.0137						12.851	0.564	0.998	4.223	0	0.0199	3.54	0	0.023083													
185												7.584	1.461	0.906																			
186	4.461	0.309	0.961	2.81	0	0.0149																											
187																							4.006	0.378	0.97	2.358	0	0.03					
189												8.733	1.055	0.921																			
190																							3.929	0.35	0.952	2.81	0	0.0226					
193	7.752	0.338	0.998	3.943	0	0.0036	4	0	0.012346			8.797	0.939	0.932																			
194												9.103	0.437	0.922	7.255	0	0.0167																
201	2.88	0.319	0.935	1.656	0	0.0399						5.577	0.447	0.952									5.269	0.318	0.995	2.606	0	0.0052	4	0	0.012346		
208												9.213	0.437	0.923	7.294	0	0.0165																
210	5.183	0.309	0.923	5.804	0	0.0177																											
212				2.81	0	0.0441																											
213																							7.19	0.352	0.978	5.413	0	0.0052					
230																							2.46	0.375	0.911								
